# Development and mechanical-functional validation of 3D-printed laparoscopic forceps

**DOI:** 10.1590/0100-6991e-20243619-en

**Published:** 2024-05-24

**Authors:** CARLOS MAGNO QUEIROZ DA CUNHA, ANA PAULA BOMFIM SOARES CAMPELO, LUCAS BUFFAT SALES, IAN BARROS LEAL MALVEIRA ARY, JOSÉ WALTER FEITOSA GOMES, MÁRCIO WILKER SOARES CAMPELO

**Affiliations:** 1 - Centro Universitário Christus, Mestrado de Inovação Tecnológica em Saúde - Fortaleza - CE - Brasil; 2 - Centro Universitário Christus, Curso de Medicina - Fortaleza - CE - Brasil; 3 - Termite Engenharia Inovativa - Fortaleza - CE - Brasil; 4 - Insituto Dr. José Frota, Serviço de Cirurgia Geral - Fortaleza - CE - Brasil; 5 - Universidade Federal do Ceará, Departamento de Cirurgia - Fortaleza - CE - Brasil

**Keywords:** Laparoscopy, Printing, Three-Dimensional, Education, Medical, Laparoscopia, Impressão Tridimensional, Educação Médica

## Abstract

**Introduction:**

3-dimensional printing has enabled the development of unique and affordable additive manufacturing, including the prototyping and production of surgical forceps. Objective: demonstrate the development, 3D printing and mechanical-functional validation of a laparoscopic grasping forceps.

**Methods:**

the clamp was designed using a computer program and printed in 3 dimensions with polylactic acid (PLA) filament and added 5 screws for better leverage. Size and weight measurements were carried out, as well as mechanicalfunctional grip and rotation tests in the laboratory with a validated simulator.

**Results:**

Called “Easylap”, the clamp weighed 48 grams, measured 43cm and was printed in 8 pieces, taking an average of 12 hours to produce. It allowed the simulation of the functional characteristics of laparoscopic pressure forceps, in addition to the rotation and rack locking mechanism. However, its strength is reduced due to the material used.

**Conclusion:**

It is possible to develop plastic laparoscopic grasping forceps through 3-dimensional printing.

## RESUMO

**Introdução:**

a impressão em 3 dimensões permitiu o desenvolvimento de manufaturas aditivas únicas e acessíveis, inclusive na prototipagem e produção de pinças cirúrgicas. Objetivo: Demonstrar o desenvolvimento, a impressão em 3D e a validação mecânico-funcional de pinça laparoscópica do tipo apreensão.

**Métodos:**

a pinça foi desenhada em programa de computador e impressa em 3 dimensões com filamento de ácido poliláctico (PLA) e acrescida de 5 parafusos para melhor efeito de alavanca. Foram realizadas aferições de tamanho e peso, bem como testes mecânicos-funcionais de preensão e rotação em laboratório com simulador validado.

**Resultados:**

denominada “Easylap”, a pinça pesou 48 gramas, mediu 43 cm e foi impressa em 8 peças, levando em média 12 horas para sua produção. Ela permitiu a simulação das características funcionais de pinça laparoscópicas de apreensão, além de mecanismo de rotação e travamento por cremalheira. Porém sua força é reduzida devido ao material utilizado.

**Conclusão:**

é possível desenvolver pinça laparoscópica plástica de apreensão através de impressão em 3 dimensões.

## INTRODUCTION

Three-dimensional (3D) printing allows additive manufacturing through digital models designed on a computer^1^ ^,^ ^2^. Its creation in the 1980s was an industrial milestone, having diversified and advanced in relation to new equipment and printing materials, ranging from plastic polymers to metals to bioprinting with cells^3^ ^,^ ^4^.

In the health field, one of the most studied uses of 3D printing is the development and plastic prototyping of surgical forceps, since this advent enables to easily customize and adapt these materials, making them lighter and more comfortable, both for the surgeon and for the patient^5^ ^,^ ^6^.

In parallel to the development of forceps, 3D printing can also innovate in medical training, as it allows the diversification of tools already used in simulators and teaching models with plastic^7^ ^-^ ^9^.

Given the mentioned benefits and the constant evolution of this technology, the present study aims to demonstrate the development, 3D printing, and mechanical-functional validation of a laparoscopic grasping forceps model called Easylap.

## METHODS

### Development and printing

**Figure 1:**
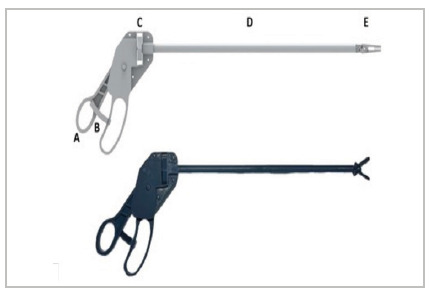
Digital design of the printed and assembled forceps.

This is a technological development study, in which the authors modeled Easylap using the Siemens’ Solid Edge software, version 2022. The product design (Figure 1) aimed at incorporating traditional models of laparoscopic grasping forceps to the specific needs for 3D printing additive manufacturing, since some characteristics of plastic polymers, such as resistance and malleability, are different from the ones of the metal commonly used in surgical forceps.

We used a 3D printer of the Fused Deposition Modeling (FDM) type and polylactic acid (PLA) filament to print the forceps.

Easylap is made up of eight parts that were printed on a single printing tray, taking on average 12 hours per forceps with the best printing quality. 

### Assembly

**Figure 2:**
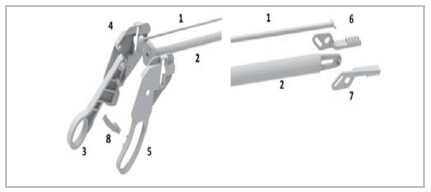
Assembly of the forceps: Part 1 is fitted inside part 2. The ball of part 1 is fitted into part 3. With this assembly formed, parts 4 and 5 are fitted laterally at the proximal end and parts 6 and 7 at the distal end. Fitting part 8 at the proximal end is optional (rack).

After printing, all parts are polished for better fit, being assembled as shown in Figure 2. In addition, five screws with five nuts measuring 1.4mm x 6mm, already printed as shown in Figure 1, are positioned in predefined insertion locations and without the need for instruments cutting tool or drill to fit them. This addition generates greater stability of the handle and, consequently, better leverage.

### Mechanical tests

First, the prototype was weighed on a precision scale, and measured with a caliper and protractor. To estimate the jaw grip strength, the forceps was placed at 0º and its jaw was attached to a digital dynamometer that was subjected to manual traction until the forceps jaw deformed. The value was recorded on the dynamometer in Kilogram-force (KgF) and manually converted into Newton (N) according to the formula: 1 KgF = 9.81 N. We carried out this test in two scenarios, the first with only the proximal end closed with the rack and the second without the rack, but with external force (human hand on the proximal end).

### Functional tests

**Figure 3:**
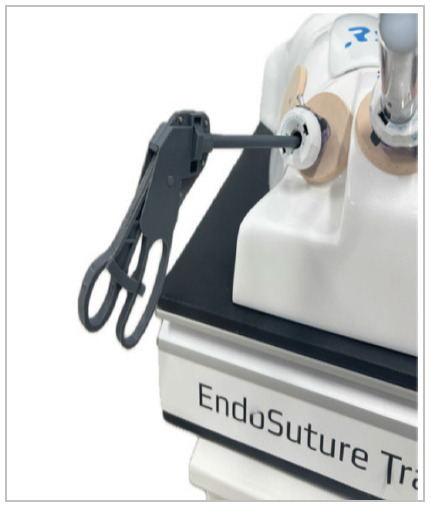
Side view of forceps inserted into a laparoscopic simulator..

**Figure 4:**
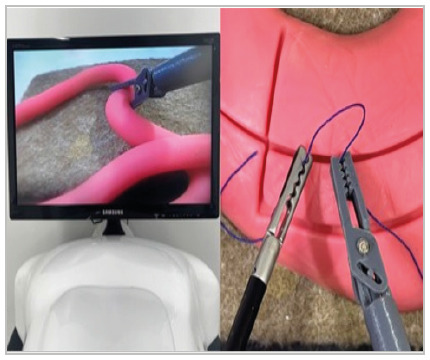
Forceps being used in a laparoscopic simulator.

Tests were performed in the laboratory using a validated abdominal cavity simulator - Endosuture Training Box^®^ (Figures 3 and 4)^10^.

The forceps were introduced into the simulator using a 10 mm trocar and four tasks were performed:

1. Moving five beans in 15 tests (75 movements) without the rack.
2. Moving five beans in 15 tests (75 movements) with the rack.
3. Moving a 100-cm tubular rubber structure that simulates intestinal loops.
4. 360º-rotation of the forceps rod using the rotation mechanism for 100 consecutive times.

## RESULTS

Assembled and screwed in, the forceps weighed 48 grams and measured 43 cm long when fully opened. Its shaft and closed jaws have a diameter of 9 mm, 30º being the maximum opening jaw angle.

With this configuration, around R$ 17.00 were spent on raw materials (PLA) and R$ 2.00 on screws and nuts, totaling R$ 19.00 in cost (around US$ 3.80 a 5.00 USD/BRL exchange rate).

The closed forceps, coupled with the rack and without the help of external force (human hand), remained with the jaw closed with a force of up to 1.71N. In the same way, but without the rack and its proximal end closed by a human hand, the jaw remained closed until a force of 2.4N was reached.

In functional tests, the forceps were able to move five beans in 15 tests without a rack and in 15 tests with a rack (150 movements in total), as well as moving the tubular rubber for 100cm without jamming (Figure 4). The rod rotation mechanism was effective, enduring 100 complete rotations without locking.

## DISCUSSION

3D printing technology has brought numerous facilities to the technology development market, including in Medicine. However, a careful and responsible approach is necessary on the part of healthcare professionals, always based on ethical and scientific principles for tests and uses within the field. Given these aspects, because our study developed the forceps using PLA, a non-sterilizable plastic filament, our application is restricted to prototyping and training in laparoscopy and cannot be used for other purposes.

From this perspective, the driving factor behind this technology and what differentiates it from training clamps produced on a large scale is the possibility of customization and adjustments according to each user in all parts, that is, the forceps can adapt to the surgeon hand, length of the training box, diameter of the trocar, among other various combinations^11^ ^,^ ^12^.

In terms of costs, plastic printing allows for countless possibilities for printers and materials, with the cost being variable and completely dependent on these factors. Therefore, for tweezers prototyping, 3D printing is an essential tool for the developer, as it allows functional tests, such as those we performed, at affordable costs^13^ ^,^ ^14^. However, for the manufacture of forceps for everyday use, the resistance and durability of metal are superior to printed plastic, and no study on the durability of PLA in laparoscopic forceps has been carried out, making a more detailed cost-benefit approach impossible.

With mechanical-functional tests, it is possible to demonstrate that the plastic impression allows simulating the functional characteristics of laparoscopic grasping forceps, in addition to the rotation and rack-locking mechanism. However, due to the limitations imposed by the material used, the resistance of the jaws is inferior to laparoscopic forceps, which reaches 8.9 N in similar tests^14^.

These results reinforce the possibility of using plastic printing for prototyping laparoscopic forceps, as well as for training in laparoscopy. However, more studies are needed to understand the impact of this mechanical difference on current teaching models.

## CONCLUSION

It is possible to develop and print plastic laparoscopic grasping forceps on a 3D printer with the same functional characteristics as commercial forceps. Nonetheless, due to the material and technique used in this study, characteristics such as strength and resistance are not equivalent to the ones of conventional forceps.
